# Addressing the Need of a Translational Approach in Peripheral Neuropathy Research: Morphology Meets Function

**DOI:** 10.3390/brainsci11020139

**Published:** 2021-01-22

**Authors:** Laura Monza, Giulia Fumagalli, Alessia Chiorazzi, Paola Alberti

**Affiliations:** 1 School of Medicine and Surgery, University of Milano-Bicocca, 20900 Monza, Italy; laura.monza@unimib.it (L.M.); giulia.fumagalli1@unimib.it (G.F.); alessia.chiorazzi@unimib.it (A.C.); 2 NeuroMI (Milan Center for Neuroscience), 20126 Milan, Italy

**Keywords:** neuropathy, neurophysiology, nerve conduction studies, EMG, animal models, neuropathology, translational medicine

## Abstract

Peripheral neuropathies (PNs) are a type of common disease that hampers the quality of life of affected people. Treatment, in most cases, is just symptomatic and often ineffective. To improve drug discovery in this field, preclinical evidence is warranted. In vivo rodent models allow a multiparametric approach to test new therapeutic strategies, since they can allow pathogenetic and morphological studies different from the clinical setting. However, human readouts are warranted to promptly translate data from the bench to the bedside. A feasible solution would be neurophysiology, performed similarly at both sides. We describe a simple protocol that reproduces the standard clinical protocol of a neurophysiology hospital department. We devised the optimal montage for sensory and motor recordings (neurography) in mice, and we also implemented F wave testing and a short electromyography (EMG) protocol at rest. We challenged this algorithm by comparing control animals (BALB/c mice) with a model of mild neuropathy to grasp even subtle changes. The neurophysiological results were confirmed with neuropathology. The treatment group showed all expected alterations. Moreover, the neurophysiology matched the neuropathological analyses. Therefore, our protocol can be suggested to promptly translate data from the bench to the bedside and vice versa.

## 1. Introduction

Peripheral neuropathies (PNs) are common conditions which affect 2–3% of the world population and can seriously interfere with daily activities and quality of life [1,2]. PNs are associated with many different pathogenetic mechanisms [3] and, frequently, effective treatments are lacking. Regardless of the cause, PNs correlate with a decrease in quality of life [4]. The search for novel treatments should start from a form of preclinical testing able to define a sound biological rationale for clinical trial design.

The advantage of preclinical testing is the possibility to perform pathogenetic studies to identify new strategies that cope with peripheral nervous system (PNS) dysfunction, but a solid readout is needed to translate the data from the bench to the bedside. Moreover, PNs comprise a variety of manifestations that are not always simple to summarize and detect, even in human subjects [5]. Despite this, nerve conduction studies (NCS) are considered the gold standard to diagnose PNs [6]. NCS in a routine clinical setting are commonly used as a first-level diagnostic tool to detect and grade the neuropathy pattern. Different from physician-based and patient-reported outcome measures, NCS can be easily reproduced with the same approach in in vivo models.

Therefore, to enhance the translation potential of preclinical testing, we devised a simple, rapid, non-invasive protocol to approach animal recordings as it is done in clinical practice. The final aim was to obtain a type of neurophysiological testing that could be the ideal companion of classical neuropathological investigations.

To best reproduce the typical protocol for PNs investigation in a hospital EMG lab, we defined the optimal montage and tested it in a model of a quite mild neuropathy, one associated with a well-known hyperexcitability syndrome (i.e., oxaliplatin (OHP)-induced peripheral neurotoxicity (OIPN)) [7].

## 2. Materials and Methods

### 2.1. Animals

Animal care and husbandry were conducted according to institutional guidelines, specifically national (D.L.vo n. 26/2014) and international laws (EEC Council Directive 86/609, OJ L 358, 1, 12 December 1987; Guide for the Care and Use of Laboratory Animals, U.S. National Research Council, 1996). Sixteen male BALB/c mice (Envigo, Udine, Italy) were employed for this study (19–21 g at arrival).

### 2.2. Study Design

Two groups were compared (*n* = 8 each): a control and a treatment group. The treatment group received OHP tail vein injections (3.5 mg/kg) twice weekly (separated by either 3 or 4 days) for 4 weeks [8]. The control group was composed of untreated mice.

NCS were performed at baseline and after chemotherapy completion, obtaining sensory recordings for the caudal and digital nerves and motor recordings and F waves for the sciatic nerve. EMG recordings at rest were performed at baseline 24 h after the first administration, mid-treatment, and the day before the last administration. All animals of both groups underwent all neurophysiological observations at each time point.

Animals were sacrificed for confirmatory neuropathological analyses at the end of the observational period (i.e., after neurophysiological assessment at the end of treatment), and samples from three animals per group were analyzed for the histophatological confirmation of neuropathy development.

### 2.3. Neurophysiology Protocol

NCS were performed with a Myto2 electromyography apparatus (ABN Neuro, Firenze, Italy). Subdermal needle electrodes were employed (Ambu Neuroline (Ambu^TM^, Ballerup, Denmark)). All the neurophysiological determinations were performed under standard conditions in a temperature-controlled room (22 ± 2 °C) and under deep isoflurane anesthesia. The optimal setting of stimulation for each nerve was reached following the subsequent protocol.

**Figure 1:**
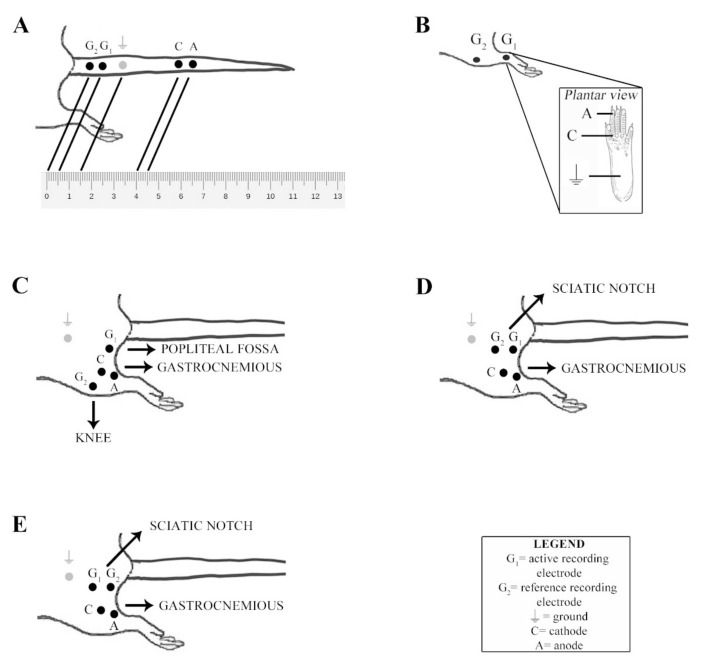
Nerve conduction study (NCS) montage. (**A**) Caudal nerve sensory conduction study (SCS) montage. (**B**) Digital nerve SCS montage. (**C**) Sciatic nerve motor conduction study (MCS) montage at the distal site of stimulation. (**D**) Sciatic nerve MCS montage at the proximal site of stimulation. (**E**) Sciatic nerve F wave montage.

The caudal nerve Sensory Conduction Study (SCS; see Figure 1A) was obtained, placing a pair of recording needle electrodes at the base of the tail (interelectrode distance: 0.5 cm) and a pair of stimulating needle electrodes (interelectrode distance: 0.5 cm) 3.5 cm distally to the active recording electrode. The ground electrode was placed 1 cm distally to the active recording electrode. For the digital nerve SCS, see Figure 1B. The positive recording electrode was placed in front of the patellar bone, the negative recording electrode was close to the ankle bone, the positive and negative stimulating electrodes were close to the fourth toe near and under the paw, respectively, and the ground electrode was placed in the sole. For the sciatic nerve Motor Conduction Study (MCS) we first performed the distal stimulation (see Figure 1C) by placing the positive and negative recording electrodes in the gastrocnemious muscle. At this point, the negative stimulating electrode was placed deep in the popliteal fossa, and the positive stimulating electrode was placed in front of the patellar bone. The ground electrode was placed in the lateral side of the animal. Then, we performed the proximal stimulation (see Figure 1D) by placing the negative stimulating electrode into the sciatic notch and the positive one at least 1 cm proximally to it, subcutaneously, in the homolateral flank of the animal (all other electrodes were left for the distal stimulation). To allow a correct motor velocity determination, the fur from the distal back and hindlimb was completely removed with an electric razor, followed by hair-removal cream (Veet ^®^ hair-removal cream, Dansom Lane, Hull, UK). Afterward, any residual cream was carefully removed with a soft gauge and water. To calculate the motor conduction velocity, the position of the negative recording electrode at the proximal and distal site of stimulation was depicted on the skin with a dermographic pen. The distance between these two points was measured with a flexible string ruler to better follow the nerve’s anatomical course. F waves were obtained by stimulating the sciatic nerve, as shown in Figure 1E. The recording and ground electrodes were placed as described for the proximal stimulation of the sciatic nerve. The negative and positive stimulating electrode position was inverted respectively to what was done for the proximal stimulation of the sciatic nerve. The intensity, duration, and frequency of stimulation were set up in order to obtain optimal results, particularly the supramaximal amplitude. An averaging technique was applied carefully and only when appropriate. The conduction velocities, amplitudes, and latencies were measured as prescribed by a procedure commonly applied in clinical practice [9]. For sensory recordings, the filters were kept between 20 Hz and 3 KHz, between 20 Hz and 2 KHz for motor recordings, and between 20 Hz and 3 KHz for F waves. The sweep was kept at 0.5 ms.

EMG recordings at rest were performed with a concentric EMG needle (Ambu™, Ballerup, Denmark). The following muscles of the left hind limb were tested: the quadriceps femoris, gastrocnemious, and flexor digitorum. Briefly, the pathological findings at rest were named according to standard clinical practice [9]:

- Augmented insertional activity (IA): any waveform other than endplate potentials at a needle movement lasting longer than 300 ms;
- Fibrillation potentials (fibs): brief spikes with an initial positive deflection, lasting 1–5 ms, a regular firing pattern, and being low in amplitude (10–100 μV);
- Positive sharp waves (PSWs): brief initial positivity followed by a long negative phase, regular firing pattern, and variable amplitude (10–100 μV, occasionally up to 3 mV);
- Fasciculation potential (fasc): a single, spontaneous, involuntary discharge of an individual motor unit that fires slowly and irregularly (less than 1–2 Hz);
- Complex repetitive discharges (CRDs): high-frequency (5–100 Hz), multi-serrated, repetitive discharges with an abrupt onset and termination;
- Myotonic discharge (MD): high-frequency (150–250 Hz), decrementing, repetitive discharges of a single motor unit [10].

All these findings were used to rate the increased motor unit activity according to the scoring system proposed by Hill et al. in a cohort of OHP-treated patients. Their score was demonstrated to have a 100% sensitivity and specificity to detect acute changes due to OHP [11]:

- Score 0: no abnormal findings at rest;
- Score 1: increased insertional activity;
- Score 2: spontaneous, high-frequency firing lasting less than 2 s;
- Score 3: spontaneous, high-frequency firing lasting 2–5 s;
- Score 4: spontaneous, high-frequency firing lasting more than 5 s.

To rate each animal at all time points, we used the worst score among the three tested muscles. The sweep was kept at 100 ms and the sensitivity at 200 µV.

### 2.4. Neuropathology

The left sciatic nerves were harvested and processed for the morphological analysis (3 animals per group). The tissues were fixed for 3 h at room temperature in 3% glutaraldehyde, post-fixed in OsO4 and embedded in epoxy resin. Morphological analysis was carried out on 1 µm thick semi-thin sections stained with toluidine blue. At least two tissue blocks for each animal were sectioned and then examined with a light microscope (Nikon Eclipse E200, Nikon Europe B.V., Amsterdam, The Nederlands).

### 2.5. Statistical Analysis

Descriptive analysis was performed for all the neurophysiological parameters. The neurophysiology data were analyzed for significant differences between groups through the Mann–Whitney U test. In all cases, a priori *p* < 0.05 was considered significant. Data analysis was carried out using the GraphPad 3.0 software (GraphPad Software, Inc., La Jolla, CA, USA).

The sample size for each experiment was calculated on the basis of the nerve conduction velocity (NCV) reference values [12], assuming that the relevant difference between the control (CTRL) and OHP groups was 5 m/s (standard deviation = 7). Thus, if a two-sided 5% alpha and an 80% power was set, the sample size was 7 animals per group. We slightly increased the number (8 animals per group) to ensure the minimum number at the end of the observational period, in case the animals had adverse reactions during treatment.

## 3. Results

### 3.1. Neurophysiology

#### 3.1.1. Nerve Conduction Studies

All the parameters obtained for the caudal, digital, and sciatic nerves were not statistically significant between the two groups at baseline, assuring homogeneity (data not shown). At treatment completion, as shown in Table 1, NCS showed a deterioration of the sensory parameters for the caudal and sciatic nerves. The motor recordings as well as the F waves were, instead, similar between the control and the OHP group.

#### 3.1.2. EMG

At baseline and throughout all the observational period, the control animals did not show any alterations for the EMG testing at rest. Conversely, the OHP-treated animals, while unaltered at baseline, showed at each subsequent time point in any tested muscle a clearly altered EMG pattern at rest, compatible with an altered excitability (see Table 2). Alterations were more prominent in most of the distal muscles (e.g., the flexor digitorum) and of a higher degree with treatment progression. The most common finding was increased IA, followed by PSWs, fibs, and fasc; CRDs and MDs were less frequent and observed mainly starting from mid-treatment.

Considering all the findings for each individual muscle, a score for the increased motor unit activity was given at each treated animal at each time point, as summarized in Table 3. As soon as after the first administration, all animals showed at least a score of 2, reaching up to a score of 3. At mid-treatment and as soon as before the last administration, the scoring was at least 3 for all animals, reaching up to the maximum score of 4; a score of 4 was present in more than 50% of the animals at the last time point.

### 3.2. Neuropathology

**Figure 2:**
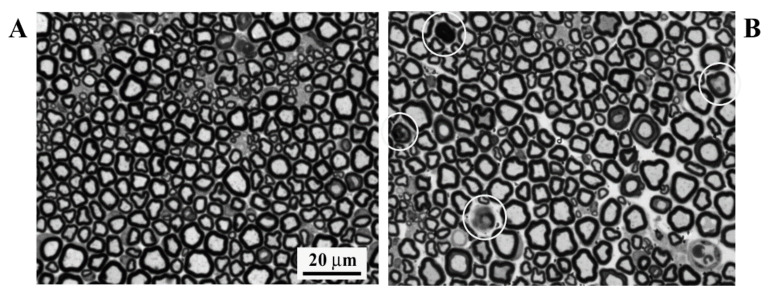
Morphological analysis of the sciatic nerve at 40× magnification. (**A**) Exemplificative image of the control group animal specimen. (**B**) Representative image of an OHP group’s findings. White circles represent the few degenerating fibers.

A confirmatory neuropathological assessment (*n* = 3/group) allowed us to verify the degree and pattern of nerve damage. In the OHP-treated animals, a very mild axonopathy with a few degenerating fibers was detected (see Figure 2).

## 4. Discussion

The proposed protocol was tested in a challenging setting: PNS alterations induced by OHP. We selected this model for two main reasons. First, OHP primarily induces neuronopathy (i.e., damage of the soma of dorsal root ganglia sensory neurons) and, subsequently, this causes axonal dysfunction via a dying back mechanism. The neuropathy pattern expected in an NCS is not a severe one, but a quite mild, sensory-only one. Second, OHP induces functional changes in axons due to a transient unbalance of voltage-operated ion channels [7,13,14], thus giving the opportunity to test the usefulness of EMG recordings in the presence of functional, transitory changes. OHP, in fact, causes an acute neurotoxicity syndrome, lasting 24–72 h after each administration and with hallmarks of axonal hyperexcitability [7,15,16,17]. Patients report cold-triggered paresthesia, cramps, spasms, and fasciculations with matching EMG changes at rest, in the absence of motor neuropathy [11,18,19].

The proposed NCS protocol tests the digital branch of the sural nerve (pure sensory), caudal nerve (mixed nerve, similar to the ulnar nerve in humans [9]), and sciatic nerve (motor). It was devised for reproducing the most common protocols to screen for PNs in patients, which comprehend a pure sensory (sural nerve), motor (peroneal or tibial nerves), and mixed nerve (ulnar nerve) at different sites (i.e., the upper and lower limbs). Moreover, the digital nerve in mice, similar to the dorsal sural nerve in humans [20], is ideal for the early detection of sensory damage of the PNS, given its anatomical characteristics (one of the most distal sensory branches in the hind limbs). Caudal nerve determinations allowed for verification of whether multisite damage occurred.

For sensory determinations, an orthodromic stimulation was performed to avoid artifacts due to motor activation (this could have been more intrusive in an antidromic setting). Notably, by using subdermal needles, the amplitude of the recorded potentials was appropriate to allow reliable measurements in all cases. The set-up for motor studies was planned carefully. The gastrocnemious muscle was the derivation site, since the course of the nerve could be measured more accurately if compared with the plantar muscles [21]. F wave responses from the same nerve were obtained, inverting the polarity of the dipole at the proximal site of stimulation, due to the reduced length of the mouse’s hind limb, without encountering any technical difficulty (clear traces were obtained in all tested animals). Regarding the EMG recordings, the muscles in the hind limb (with a proximal to distal distribution) were tested in the same limb where no motor neuropathy was observed in the NCS.

The NCS results, obtained at the end of the observational period, proved that the algorithm was able to detect all expected changes, as known from clinical practice, as a consequence of OHP administration [7,22,23,24]: a mild sensory axonal neuropathy in the absence of changes in motor neuropathy. Moreover, the mild, axonal pattern of damage was confirmed via neuropathological analysis.

EMG monitoring was informative at all time points. At baseline, no alterations were present, as expected, whereas the monitoring throughout the whole treatment evidenced increased muscle excitability. Increased IA (a sign that an underlying neuropathic or myopathic condition is ongoing), in fact, was the most common observation, but it was associated with many others, which is suggestive of altered excitability. The fibs and PSW, as well as the MD, were due to the spontaneous depolarization of a muscle fiber. The CRDs were due to the depolarization of a single muscle fiber, followed by ephaptic spread to adjacent denervated fibers; the fascs were single, spontaneous, involuntary discharges of an individual motor unit [9]. Since the first administration, data were similar to the ones described in OHP patients by Hill et al. [11], Wilson et al. [18], and Lehky et al. [19].

The aim of the experiment was not a formal assessment of acute OIPN, since EMG recordings at rest are just a qualitative, indirect measure of it. Nevertheless, the EMG results were in line with recent findings by our group, obtained via nerve excitability testing in rodents [17], as well as by other groups in clinical [13,25] and preclinical settings [26,27]. Even if it was a qualitative approach, we were able to score our assessment, observing a progression of the pattern along the month of treatment. Therefore, we can conclude that the proposed protocol was able to grasp even subtle changes in EMG, even in the absence of damage to the motor system.

The algorithm we proposed can be considered more comprehensive in respect to the others previously described. In the vast majority, only the motor system (most frequently one nerve) [28,29,30,31,32,33,34,35,36,37,38,39,40,41,42,43] or only the sensory or mixed nerves [12,44,45,46,47] were tested. A few groups tested both the motor and sensory systems, but none of the tests were performed at the same time as EMG recordings [48,49,50,51,52,53,54].

There are some limitations to be acknowledged. Data from a single experiment were presented, and a single disease model was used (very mild sensory axonal neuropathy, plus hyperexcitability syndrome). However, it can be reasonably stated that the presented methods can be adequately reproduced in more complex and severe settings. Moreover, the decision to detect EMG alterations, in the absence of motor neuropathy, allowed to verify its appropriateness even for subtle changes. The sample size, despite being low, was at least one unit higher than the minimum number required for neurophysiology (which was the primary endpoint for neuropathy assessment). Neuropathological confirmatory analyses were performed on three animals per group, but this relatively low number per group is adequate, given the fact that this very mild model of axonopathy was well characterized previously [12].

## 5. Conclusions

We presented a neurophysiological protocol that reproduces in mice what is routinely done in any hospital EMG lab. OIPN, in which axonal damage is expected to be modest, was the test bench for the proposed algorithm. OHP does not cause motor neuropathy, but only a sensory axonal neuropathy and an axonal and muscular hyperexcitability syndrome. Thus, it was verified if the EMG recordings at rest were able to detect even the latter very mild condition.

In conclusion, our non-invasive, simple, rapid (10 min/animal) neurophysiological protocol can be suggested as the ideal companion of morphological analyses and as the translational outcome measure in PNS disorder drug discovery.

## Figures and Tables

**Table 1:** NCS values after treatment completion and Mann–Whitney U test results.

Parameter	Control Group (Median, LL ^1^, and UL ^2^ of 95% CI ^3^)	OHP Group (Median, LL, and UL of 95% CI)	Mann–Whitney U Test
Caudal nerve SNAP amplitude (μV)	125.1 (110.3–152.4)	75.70 (50.04–113.7)	**<0.05**
Caudal nerve sensory conduction velocity (m/s)	32.10 (31.39–32.69)	28 (27.16–29.32)	**<0.0005**
Digital nerve SNAP amplitude (μV)	124.5 (108.2–144.0)	60.40 (46.37–80.98)	**<0.0005**
Digital nerve sensory conduction velocity (m/s)	26.50 (25.22–27.43)	25.00 (24.37–25.30)	**<0.05**
Sciatic nerve CMAP amplitude (mV)	72.95 (66.40–78.78)	65.80 (58.71–75.01)	n.s. ^4^
Sciatic nerve motor conduction velocity (m/s)	53.45 (49.69–57.42)	62.20 (53.90–65.75)	n.s.
F waves (sciatic nerve, m/s)			n.s.

^1^ Lower limit. ^2^ Upper limit. ^3^ Confidence interval. ^4^ Not significant. CMAP: Compound Muscle Action Potential. SNAP: Sensory Nerve Action Potential.

**Table 2:** EMG findings for animals treated with oxaliplatin (OHP) at different time points.

Animal	Muscle	Before First Administration	After First Administration	At Mid-Treatment	Before Last Administration
01	Gastrocnemious	none	IA, fibs 1+, PSW 1+, CRD	IA	IA, CRD
	Quadriceps	none	IA	IA, PSW 1+	IA
	Flexor digitorum (hind limb)	none	IA, PSW 2+, CRD	IA	IA, PSW 1+
02	Gastrocnemious	none	IA, fibs 1+, PSW 1+	IA, PSW 1+	IA, CRD
	Quadriceps	none	IA	IA, PSW 1+	IA, PSW 1+
	Flexor digitorum (hind limb)	none	IA, fibs 1+	IA, MD	IA, PSW 1+
03	Gastrocnemious	none	IA, fibs 1+	IA	IA, PSW 1+, fasc 1+, fibs 1+
	Quadriceps	none	IA	IA	IA, PSW 1+
	Flexor digitorum (hind limb)	none	IA	IA	IA, PSW 1+, fasc 1+, CRD
04	Gastrocnemious	none	IA, fibs 1+, PSW 1+	IA, PSW 1+	IA, PSW 3+, CRD
	Quadriceps	none	IA, fibs 1+, PSW 1+, fasc 1+	IA, PSW 1+	IA, fibs 1+
	Flexor digitorum (hind limb)	none	IA, fibs 1+, PSW 1+	IA, PSW 2+	IA, fibs 1+, fasc 1+
05	Gastrocnemious	none	IA, PSW 1+	IA, PSW 1+	IA, PSW 1+, fibs 1+
	Quadriceps	none	IA	IA, PSW 1+, fibs 1+	IA, PSW 1+, fibs 1+
	Flexor digitorum (hind limb)	none	IA, PSW 1+	IA, PSW 1+	IA, PSW 1+, fibs 1+, CRD
06	Gastrocnemious	none	IA, fibs 1+, PSW 2+	IA, PSW 1+	IA, PSW 2+, CRD, MD
	Quadriceps	none	IA, fibs 1+, PSW 2+	IA, PSW 2+	IA, PSW 2+
	Flexor digitorum (hind limb)	none	IA, fibs 1+, fasc 1+, PSW 2+, CRD	IA, PSW 1+, CRD, MD	IA, PSW 1+, CRD, MD
07	Gastrocnemious	none	IA, PSW2+, fibs 2+, CRD	IA, PSW 2+	IA, PSW 2+, CRD
	Quadriceps	none	IA	IA, PSW 2+	IA, PSW 2+
	Flexor digitorum (hind limb)	none	IA, PSW 1+, CRD	IA, PSW 1+, fibs 1+, MD	IA, PSW 1+, CRD, MD
08	Gastrocnemious	none	IA, PSW2+, CRD	IA, PSW 2+, fibs 1+	IA, PSW 1+, CRD
	Quadriceps	none	IA	IA, PSW 2+	IA, PSW 1+, fibs 1+
	Flexor digitorum (hind limb)	none	IA, PSW+, MD	IA, PSW 1+, fibs 1+	IA, PSW 1+, fibs 1+, fasc 1+

CRD: complex repetitive discharge; fasc: fasciculation potentials; fibs: fibrillation potentials; MD: myotonic discharge; PSW: positive sharp waves; IA: augmented insertional activity. For fasc, fibs, and PSW, this scoring system was applied: 1+ for persistent single trains of potentials (>2–3 s) in at least two areas; 2+ for a moderate number of potentials in three or more areas; 3+ for many potentials in all areas; and 4+ for a full interference pattern of potentials.

**Table 3:** Increased motor unit activity for each animal at a different time point.

Animal	Before First Administration	After First Administration	At Mid-Treatment	Before Last Administration
01	0	2	3	3
02	0	2	3	3
03	0	2	2	4
04	0	3	3	3
05	0	3	3	4
06	0	3	4	4
07	0	3	4	4
08	0	3	4	4

## Data Availability

Data will be made available upon request to the corresponding author.
